# Using Poly(amidoamine) PAMAM-βCD Dendrimer for Controlled and Prolonged Delivery of Doxorubicin as Alternative System for Cancer Treatment

**DOI:** 10.3390/pharmaceutics16121509

**Published:** 2024-11-23

**Authors:** Kendra Sorroza-Martínez, Ignacio González-Sánchez, Raúl Villamil-Ramos, Marco Cerbón, Jorge Antonio Guerrero-Álvarez, Cristina Coronel-Cruz, Ernesto Rivera, Israel González-Méndez

**Affiliations:** 1 Departamento de Sistemas Biológicos, Unidad Xochimilco, Universidad Autónoma Metropolitana, Calzada del Hueso 1100, Col. Villa Quietud, Mexico City CP 04960, Mexico; ksorroza@correo.xoc.uam.mx; 2 Departamento de Biología, Facultad de Química, Universidad Nacional Autónoma de México, Circuito Escolar, Ciudad Universitaria, Mexico City CP 04510, Mexico; igonzalez@quimica.unam.mx (I.G.-S.); macer@unam.mx (M.C.); 3 Centro de Investigaciones Químicas, Instituto de Investigación en Ciencias Básicas y Aplicadas, Universidad Autónoma del Estado de Morelos, Av. Universidad 1001, Col. Chamilpa, Cuernavaca CP 62209, Mexico; raul.villamil@uaem.mx (R.V.-R.); jguerrero@uaem.mx (J.A.G.-Á.); 4 Departamento de Biología Celular y Tisular, Facultad de Medicina, Universidad Nacional Autónoma de México, Circuito Escolar, Ciudad Universitaria, Mexico City CP 04510, Mexico; cristina.coronel.c@gmail.com; 5 Departamento de Reología, Instituto de Investigaciones en Materiales, Universidad Nacional Autónoma de México, Circuito Exterior, Ciudad Universitaria, Mexico City CP 04510, Mexico

**Keywords:** PAMAM dendrimer, doxorubicin, breast cancer, inclusion complex, β-cyclodextrin, controlled release, drug delivery

## Abstract

**Background/Objectives:** Doxorubicin (Dox) is an anticancer drug used in the treatment of a wide range of solid tumors; however, Dox causes systemic toxicity and irreversible cardiotoxicity. The design of a new nanosystem that allows for the control of Dox loading and delivery results is a powerful tool to control Dox release only in cancer cells. For this reason, supramolecular self-assembly was performed between a poly(amidoamine) (PAMAM) dendrimer decorated with four β-cyclodextrin (βCD) units (PAMAM-βCD) and an adamantane–hydrazone–doxorubicin (Ad-h-Dox) prodrug. **Methods:** The formation of inclusion complexes (ICs) between the prodrug and all the βCD cavities present on the surface of the PAMAM-βCD dendrimer was followed by ^1^H-NMR titration and corroborated by 2D NOESY experiments. A full characterization of the supramolecular assembly was performed in the solid state by thermal analysis (DSC/TGA) and scanning electron microscopy (SEM) and in solution by the DOSY NMR technique in D_2_O. Furthermore, the Dox release profiles from the PAMAM-βCD/Ad-h-Dox assembly at different pH values was studied by comparing the efficiency against a native βCD/Ad-h-Dox IC. Additionally, in vitro cytotoxic activity assays were performed for the nanocarrier alone and the two supramolecular assemblies in different carcinogenic cell lines. **Results:** The PAMAM-βCD/Ad-h-Dox assembly was adequately characterized, and the cytotoxic activity results demonstrate that the nanocarrier alone and its hydrolysis product are innocuous compared to the PAMAM-βCD/Ad-h-Dox nanocarrier that showed cytotoxicity equivalent to free Dox in the tested cancer cell lines. The in vitro drug release assays for the PAMAM-βCD/Ad-h-Dox system showed an acidic pH-dependent behavior and a prolonged profile of up to more than 72 h. **Conclusions:** The design of PAMAM-βCD/Ad-h-Dox consists of a new controlled and prolonged Dox release system for potential use in cancer treatment.

## 1. Introduction

Through the years, cancer continues to be one of the main causes of death worldwide, and part of this morbidity is due to associated difficulties during its treatment. Currently, anticancer therapy relies heavily upon the administration of small molecules, which are cytotoxic drugs that attack both cancerous and noncancerous cells due to the limited selectivity of the drugs and widespread distribution of cytotoxic molecules throughout the body [1,2,3]. Chemotherapeutics are traditionally administered as slow intravenous infusions of free drugs; nevertheless, this strategy does not offer any guarantee to reduce toxicity [4]. Doxorubicin (Dox), a well-known anticancer drug, is a powerful anthracycline used in the treatment of a wide range of solid tumors, lymphomas, leukemias and breast cancer [5]. Like the rest of the chemotherapeutics, Dox has a widespread biodistribution that causes systemic toxicity; however, the main preoccupying drawback is the irreversible cardiotoxicity caused by cumulative doses after intravascular (IV) administration, leading to poor therapeutic results [6,7,8,9]. So far, the closest strategy to lessen the effects of Dox cardiotoxicity are the liposomes Doxil/Caelyx and Myocet; however, they have several drawbacks [10]. Firstly, they are known to release drugs along a concentration gradient, leading to drug release in plasma and non-target tissues [11]. Also, the liposomes’ composition has also been associated with hypersensitivity reactions [12]. Finally, liposome dispersions are statistical mixtures of particles with different size and composition, and hence, from a regulatory and manufacturing standpoint, they are not as well defined as reproducible molecules [13].

In order to improve the antitumor efficacy and systemic toxicity of existing chemotherapeutic drugs, the targeted delivery of drugs can be performed by incorporating them into an appropriate carrier system. Thus, drug delivery systems can improve the deficiencies of chemotherapeutic treatment by modifying the biodistribution and pharmacokinetics of the drug in vivo [3]. Some tumor phenomena can be exploited, like the poor perfusion of the tumor, arteriovenous shunting, necrotic and hypoxic areas, and high interstitial fluid pressure, which work against favorable drug uptake. So, targeted drug delivery systems using long-circulating nanocarriers between 20 and 200 nanometers (nm) in size, hold immense potential to improve cancer treatment, providing selectively therapeutical effective drug concentrations at the tumor site, through an enhanced permeability and retention (EPR) effect, while reducing undesirable side effects [3,14,15].

Dendrimers stand out as hyperbranched nanosystems which can be developed with high structural monodispersity and precise control over the surface structure [16,17]. With this concept in mind, PAMAM dendrimers have received lots of attention as an alternative delivery system to liposomes for the delivery of chemotherapeutics [13]. For many years, PAMAM dendrimers have been questioned for having no representative clinical applications in the chemotherapy area [18]. One of the proposed strategies to convert these dendrimers into successful drug delivery systems is the masking of the primary amines (expressed as cationic charges) to avoid the strong capacity of binding to non-target tissues and to avoid their eventual cytotoxicity in in vitro assays [19,20]. In contrast to unconjugated dendrimers, surface-modified dendrimers show more favorable toxicological and biopharmaceutical properties and, hence, are more suitable candidates as drug delivery vectors [21,22]. In this regard, higher-generation dendrimers have apparently been the most studied since they have improved the capacity of drug solubilization when compared to lower-generation dendrimers, due to the larger void volume available for drug encapsulation [23]. However, it is important to consider the strict pH conditions of the solutions to achieve drug loading [24], which in all cases must have long-term stability to maintain the drug–dendrimer complex and achieve drug delivery to the target. For this reason, when Dox is encapsulated within a classic dendrimer structure, there is no precise and reproducible control of the drug loading process, and it is accompanied by a fast release of the therapeutic agent, and all these disadvantages prevent the therapeutic applicability of this drug association method.

In this way, another option that can be used to control the therapeutic load in the dendrimer structure is to associate the chemotherapeutic drug with the dendrimer surface through a covalent linker; however, this strategy always depends on factors such as the stability of the selected linker [25], which can result in a very fast or very slow Dox release to demonstrate a therapeutic advantage over the administration of free Dox.

**Figure 1:**
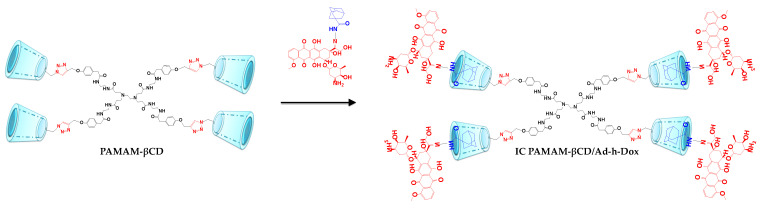
Illustration of PAMAM-βCD/Ad-h-Dox assembly formation.

As aforementioned, our research group focused on the design of a PAMAM G0 dendrimer decorated with four βCD units in the periphery (PAMAM-βCD). This modification allows the low-generation dendrimer to load guests in a controlled manner. In a previous paper [26], we reported on the physicochemical properties of this dendrimer, which include its outstanding aqueous solubility and the complete availability of βCD cavities if a suitable host is used. Furthermore, the in vitro behavior of an adamantane–hydrazone–Dox (Ad-h-Dox) prodrug was studied as a powerful tool to control Dox release only with the adequate pH stimuli [27,28,29]. Hence, in the present work, we assembled the PAMAM-βCD dendrimer with the prodrug Ad-h-Dox in order to demonstrate that a low-generation dendrimer can be effectively employed as a drug nanocarrier (Figure 1).

To demonstrate the usefulness of our design for encapsulation at the periphery of a low-generation dendrimer, we studied the controlled delivery of Dox at different pH values comparing the efficiency against a native βCD/Ad-h-Dox inclusion complex (IC). Furthermore, to determine the efficacy of the proposal nanoplatform, we assayed its in vitro cytotoxic activity for the nanocarrier alone and the PAMAM-βCD/Ad-h-Dox supramolecular assembly in different carcinogenic cell lines in order to demonstrate the advantages that this novel proposal of low-generation dendrimers can provide for the design of drug delivery systems.

## 2. Materials and Methods

### 2.1. Materials

All reagents and solvents were purchased from Merck Sigma-Aldrich Mexico and used as received, including an unmodified PAMAM G0 dendrimer, doxorubicin hydrochloride (Dox·HCl) and βCD hydrate. The synthesis of the Ad-h-Dox prodrug and PAMAM-βCD was carried out according to the methods previously published in the literature without modifications [27,28,29]. The Spectrum^TM^ Spectra/Por 6 pre-wetted standard RC dialysis tubing (molecular cut-off of 2 kD) was purchased from Fisher Scientific-Mexico.

### 2.2. NMR Experiments

Deuterium oxide (D_2_O) with isotopic purity 99.9% was obtained from Cambridge Isotope Laboratories, Inc., Mexico. No internal standard was added in order to exclude any interactions with βCD, if any. ^1^H and 2D NOESY experiments were conducted at 298 K on a Bruker Avance 500 MHz spectrophotometer. Chemical shifts are reported in ppm (δ). Multiplicities are reported by using the following abbreviations: s = singlet; d = doublet; t = triplet; br = broad and m = multiplet. ^1^H DOSY (Diffusion-Ordered SpectroscopY) NMR experiments were performed using a stimulated echo sequence incorporating bipolar gradient pulses. The gradient strength was linearly increased with 25 points from 25% up to 95% of the maximum gradient strength.

### 2.3. Job Plot Method

The Job plot method was carried out according to the previously reported methodology with some modifications [26]. Two stock solutions, (Solution Host, Sol. H) of PAMAM-βCD 2.5 mmol/L in βCD cavities and (Solution Guest, Sol. G) of Ad-h-Dox 2.5 mmol/L, were prepared in D_2_O. A series of nine samples in NMR tubes containing both PAMAM-βCD and Ad-h-Dox with a total concentration ([Ad-h-Dox] + [PAMAM-βCD]) fixed at 2.5 mmol/L were prepared. This was accomplished by introducing increasing portions of 50 μL up to 500 μL of Sol H in the adequate NMR tube, and then in the corresponding tube, decreasing amounts were added starting from 500 μL up to 50 μL of Sol G. Thus, solutions with constant volume at various βCD molar fractions (XCD = [PAMAM-βCD]/([PAMAM-βCD] + [Ad-h-Dox])) in a complete range (0.1 < r < 0.9) were obtained. The tubes were submitted to ^1^H-NMR analysis, with D_2_O as the internal standard. The continuous variation in the ^1^H-NMR chemical shift change “Δδ × XCD” (Δδ taken for adamantyl H-γ, see Figure S1 in Supplementary Information (SI), and for PAMAM-βCD/Ad-h-Dox assembled) was plotted against “XCD”.

### 2.4. Formation of Inclusion Complexes (ICs) Between βCD and PAMAM-βCD with Ad-h-Dox Prodrug

The formation of host–guest ICs between βCD and PAMAM-βCD with Ad-h-Dox was conducted according to the procedures previously reported [29,30]. A MeOH solution of Ad-h-Dox was added to the aqueous solution of PAMAM-βCD and βCD with adequate stoichiometry under vigorous stirring. After 24 h in dark conditions, the resulting translucent solution was filtered using a 0.45 μm membrane and lyophilized to give the corresponding supramolecular assembly.

In order to corroborate IC formation, the physical mixture of Ad-h-Dox, βCD and the PAMAM-βCD dendrimer was obtained by mixing the prodrug and hosts at an adequate proportion in a ceramic mortar until the formation of a uniform mixture.

### 2.5. Characterization

#### 2.5.1. Dynamic Light Scattering (DLS)

DLS experiments were carried out in water using a Zetasizer NanoS instrument at a scattering angle of 173° with a viscosity value of 0.89 mPa.s at 25 °C and a refractive index of 1.33. All samples for DLS were filtered through Milli-Q membrane filters (0.45 µm pore size) prior to measurements. Quintuplicate measurements were performed for all samples in a quartz cell. The experiments were analyzed in Zetasizer Software 7.13 version.

#### 2.5.2. Differential Scanning Calorimetry (DSC) and Thermogravimetric Analysis (TGA)

DSC analyses were performed on a Universal V4.5A TA Instruments USA. A total of 5.00 ± 0.05 mg of each sample was placed in aluminum crimped pans. The heating rate was 10 °C/min, going from 20 to 350 °C under nitrogen atmosphere (N_2_, 50.0 mL/min). A TGA was conducted on a TGA Q5000 Instrument Modulated (New Castle, DE, USA) under nitrogen atmosphere (N_2_, 10.0 mL/min) from 30 to 600 °C on a platinum pan.

#### 2.5.3. Scanning Electron Microscopy (SEM)

The surface morphology of the PAMAM-βCD dendrimer, Ad-h-Dox prodrug, their supramolecular ICs and the corresponding physical mixtures (PMs) was examined using a scanning electron microscope (SEM; JSM-7600F, JEOL Ltd., Tokyo, Japan). The samples were attached onto carbon tabs (double-sided adhesive tape) stuck to aluminum stumps. Specimens were covered with gold (plasma deposition technique) for 45 s. Pictures were taken at an excitation voltage of 15 kV.

### 2.6. In Vitro Release

The in vitro release of Dox from the assemblies PAMAM-βCD/Ad-h-Dox, βCD/Ad-h-Dox and free Dox was evaluated using a dialysis technique using a Spectra/Por 6 TRUMP-LABS membrane in phosphate-buffered saline (PBS) at pH 7.4 and citrate buffer solutions at pH = 4.5 and 3.5 as release media in order to simulate the cancerous microenvironment (pH < 6) [27]. The system was stirred at 100 rpm at 37.0 ± 0.5 °C and 3 mL; aliquots were taken at time intervals of 1, 3, 5, 7, 9, 12, 24, 48, 72, 90 and 110 h. After each withdrawal, the medium was replaced with an equal volume of fresh release medium for analysis. The content of Dox release was studied by UV-vis spectroscopy at λ = 480 nm. The release profiles were repeated in triplicate, and the outcomes were the mean value of three samples.

### 2.7. Biological Procedures

#### 2.7.1. Cell Culture

The human cancer cell lines MCF-7 and MDA-MB-231 (breast), HeLa (cervical adenocarcinoma), K-562 (myelogenous leukemia), SW-620 (colon adenocarcinoma) and SK-LU-1 (lung adenocarcinoma) were selected for cytotoxic activity studies and purchased from the ATCC^®^ (Manassas, VA, USA). Cell lines were grown in DMEM with 10 mM of non-essential amino acids, supplemented with 10% heat-inactivated fetal bovine serum (BioWest, Riverside, MO, USA). These five adherent epithelial cell lines were maintained in proliferation in a humidified atmosphere containing 5% CO_2_ at 37 °C.

#### 2.7.2. Cytotoxicity Studies

To determine the cytotoxic activity of Dox, Ad-h-Dox, the PAMAM-βCD dendrimer and all the supramolecular inclusion complexes in the selected cell lines, a tetrazolium salt-based colorimetric assay was used. Each of these cell lines was seeded in a 96-well plate at a density of 6.5 × 10^3^ cells/well at a volume of 200 μL of medium. After 24 h, the cells formed a monolayer, and 50 μL of medium was added with different concentrations of Dox, Ad-h-Dox, the PAMAM-βCD dendrimer and the corresponding supramolecular assembles according to the cell line. The treated cells were cultured in six wells by concentration, and the experiments were performed three times independently. Cell viability was determined after 48 h by adding 10 μL of 2.5 mg/mL 3-(4,5-dimethylthiazol-2-yl)-2,5-diphenyltetrazolium bromide (MTT). Insoluble formazan, produced during an incubation period of 2 h at 37 °C, was measured. The supernatant was removed, and the formazan crystals were solubilized by adding 150 µL of DMSO. The absorbance of each well was determined at 540 nm, using an Epoch Microplate reader (BioTek Instrumentals, Inc., Winooski, VT, USA). Absorbance was directly related to % cell viability, and control cells without treatment were considered to have 100% viability. The half-maximal inhibitory concentration (IC_50_) for each treatment as a cytotoxicity parameter was determined by nonlinear curve fitting from a plot of concentration versus inhibition data using Origin 7.0 (OriginLab Corporation, Northampton, MA, USA).

## 3. Results and Discussion

### 3.1. Characterization of βCD/Ad-h-Dox as Positive Control for IC Formation

In order to carry out a correct follow-up that would allow us to demonstrate that the formation of the supramolecular assembly between the PAMAM-βCD nanosystem and the prodrug Ad-h-Dox was carried out with the four cavities of the βCD present on the surface of the PAMAM dendrimer, a simple system was started by analysis, so the IC between βCD and the Ad-h-Dox prodrug was characterized, and this supramolecular assembly was taken as a positive control. In addition, this native system was previously reported on [29]. Figure S2 in the SI shows the ^1^H-NMR spectrum where the signals of the aromatic protons b, a and c of anthracycline residue are observed (between 7.5 and 7.0 ppm), and at 5.38 ppm, the signal of the proton h of the residue that links anthracycline with daunosamine (dau) sugar appears. Then, at 4.92 ppm, the anomeric proton H-1 of βCD and the aliphatic protons n, j, l, k, d and g (between 5.5 and 2.6 ppm) of dau residue appear, followed by protons H-3 and H-5 situated in the internal cavity of βCD (between 3.76 and 3.74 ppm) overlapped with proton H-6 of the primary face of βCD at 3.74 ppm. Between 3.62 and 3.45 ppm, the protons H-2 and H-4 of the same macrocycle appear. At up-field shift, the appearance of protons α–γ for the adamantane (Ad) ring (between 2.3 and 1.6 ppm) stands out. Thus, these α–γ protons are precisely the ones that interact with the H-3 and H-5 protons located in the internal βCD cavity to verify the self-assembly process. In fact, these signals overlapped with the signals of the methylene groups i, e and f present in the native Dox structure. At 1.22 ppm, another important signal appears from the methyl group (d) present in the dau residue of the Dox structure. To corroborate through-space interactions between the host and guest, the 2D NOESY NMR spectrum (see Figure S3 in SI) of the IC βCD/Ad-h-Dox was recorded, and we can observe that the protons of the Ad ring present in the Ad-h-Dox structure interact in the space with protons H-3 and H-5 present in the internal cavity of βCD, thus making the formation of the supramolecular assembly evident. Once this system is identified, the most significant findings for the PAMAM-βCD/Ad-h-Dox nanocarrier can be obtained and are detailed in the following sections.

#### 3.1.1. Job Plot Method for Dendrimer PAMAM-βCD/Ad-h-Dox

**Figure 2:**
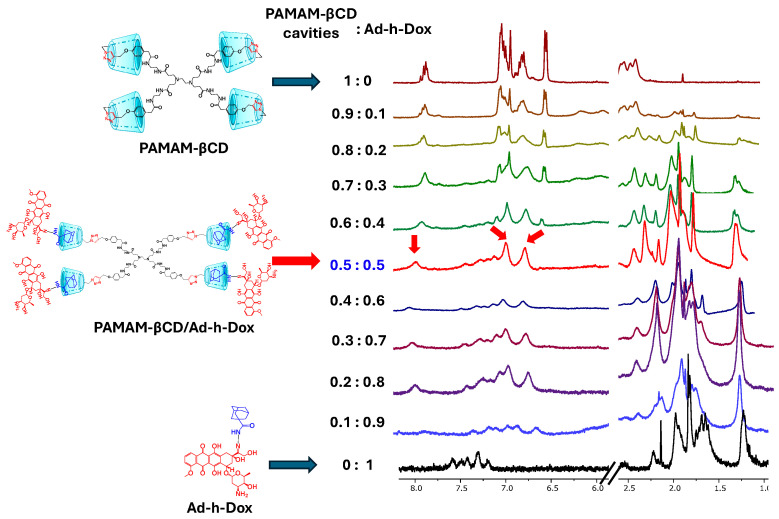
^1^H-NMR titration experiment for PAMAM-βCD dendrimer with Ad-h-Dox prodrug. In this figure, 8.2–5.5 ppm and 2.5–1.0 ppm regions are shown for mixtures of PAMAM-βCD and Ad-h-Dox in D_2_O with decreasing molar fraction from top to bottom, expressed with respect to concentration in βCD cavities for PAMAM-βCD (XCD = [βCD]/([βCD] + [Ad-h-Dox]). Total concentration [βCD] + [Ad-h-Dox] = 2.5 mM.

To demonstrate the stoichiometry of IC formation between PAMAM-βCD and the Ad-h-Dox prodrug, Job’s plot analyses were performed (see Figure 2). Based on our previous experiences with ^1^H NMR characterization, we started by identifying the structures of the PAMAM-βCD nanocarrier in D_2_O formed by the tumbling process (spectrum 1:0 in Figure 2), previously described by our research group [26]. Furthermore, even in the presence of inverted βCD cavities in the starting water solutions, it was possible to make them available for the inclusion phenomena, thanks to a shift in the tumbling process by using a strongly related guest [26], in our case, the Ad residue that is linked by the hydrolysable hydrazone bond at acidic pH to the Dox structure.

Thus, once the 0.5:0.5 molar ratio was reached, it was noticeable that the signal of the triazole proton appears as a singlet at 7.94 ppm and that of the phenyl protons as two doublets at 7.06 and 6.82 ppm. This was interpreted as the total disappearance of the reported tumbling form of βCD units and consequently the IC formation with the four βCD cavities present on the PAMAM-βCD surface.

#### 3.1.2. ^1^H-NMR and 2D NOESY Characterization of IC PAMAM-βCD/Ad-h-Dox

**Figure 3:**
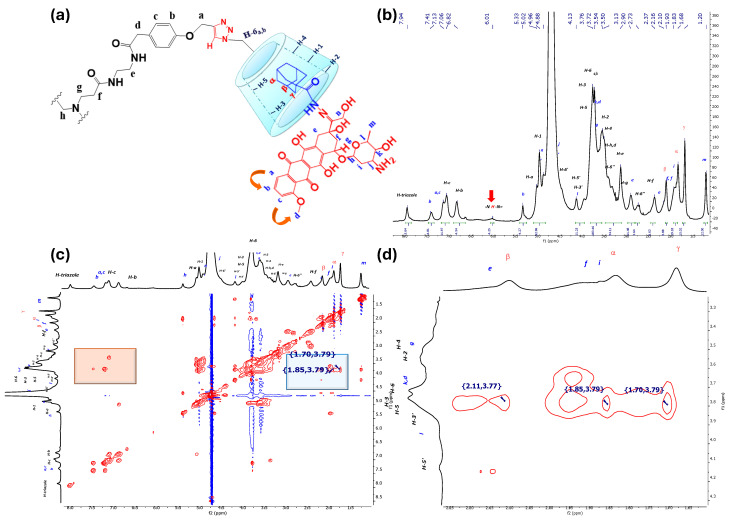
NMR characterization for nanocarrier PAMAM-βCD/Ad-h-Dox, (**a**) assignment for PAMAM-βCD dendrimer and Ad-h-Dox prodrug assemblies, (**b**) ^1^H-NMR spectra of PAMAM-βCD/Ad-h-Dox in D_2_O, (**c**) 2D NMR NOESY spectrum of IC PAMAM-βCD/Ad-h-Dox in D_2_O (highlighted in blue is interaction between protons from Ad residues with inner cavity in βCD units and in orange their intramolecular interactions), (**d**) amplification of aliphatic zones in NMR NOESY spectrum for IC PAMAM-βCD/Ad-h-Dox.

Based on the previous characterization for the positive control of the IC βCD/Ad-h-Dox, we started with an adequate assignment for the PAMAM-βCD/Ad-h-Dox assembly (see Figure 3a) in order to identify the signals of the nanocarrier and their guest Ad-h-Dox. Thus, as described above, a single signal was observed at 7.94 ppm, corresponding to the proton of the triazole ring (see Figure 3b), which demonstrates that these four rings are equivalent, and all the βCD cavities present in the PAMAM-βCD nanocarrier form an IC with the Ad-h-Dox prodrug.

In agreement with the analysis performed for the IC βCD/Ad-h-Dox, the signals of aromatic protons b, a and c appear at 7.41–7.13 ppm for the anthracycline residue. Moreover, to support the fact that the tumbling process completely disappeared after the IC formed between βCD units and Ad-h-Dox prodrugs, the signals at 7.06 and 6.82 ppm belong to *para*-substituted aromatic protons that are part of the PAMAM-βCD nanocarrier. At 6.01 ppm, it is possible to identify a broad signal of the proton of the hydrazone linker that is spatially outside the cavity of the βCD units [29]. The rest of the proton signals for βCD units appear in the order described above with the addition of the signals corresponding to the protons of the arms of the dendrimer. Finally, between 2.10 ppm and 1.68 ppm, the β, α, γ classical signals of the Ad ring protons appear, which allow us to track the self-assembly phenomenon with the βCD cavity. In this regard, in Figure 3c,d, the observed NOE interactions confirm the assembly with the internal cavity of βCD through the interactions of protons β, α and γ with the internal protons H-3 and H-5 of βCD.

It is worth mentioning that the orientation of IC formation between the Ad-h-Dox prodrug with the βCD cavities in the nanocarrier is influenced exclusively by the Ad residue [29,30,31], and there is no competition between the latter and the anthracycline ring. This assumption can be confirmed by a careful analysis of the 2D NOESY spectrum in Figure 3c, since no interactions of protons b, a or c with protons H-3 and H-5 of the inner βCD cavity are observed, and only neighboring interactions between themselves can be perceived (see part highlighted in orange in Figure 3c).

Additionally, ^1^H DOSY NMR is a powerful and versatile NMR method for the analysis of supramolecular systems, including host–guest ICs which allow for the tracking of the formation of a single host–guest system [32,33,34,35]. So, we started with the analysis for nanocarrier PAMAM-βCD (see Figure S4 in SI), and in Table 1, their diffusion coefficient values are presented compared to the diffusion coefficient of the previously reported PAMAM-βCD/AdCOOH assembly [26], which shows a small change (0.5 10^−10^ m^2^/s) with respect to the nanocarrier alone and under the premise that guest AdCOOH is completely included in the βCD cavity [26].

Then, for the PAMAM-βCD/Ad-h-Dox assembly, the diffusion coefficient was determined (see Table 1 and Figure S5 in SI), and this value reveals the formation of a single host–guest system taken as a reference in previous studies for PAMAM-type nanoconjugates [36], so we can confirm that no unbound Ad-h-Dox molecules or other dendrimeric structures (i.e., containing less than four complexed Ad-h-Dox molecules) were found in the sample.

#### 3.1.3. DLS Analysis

**Figure 4:**
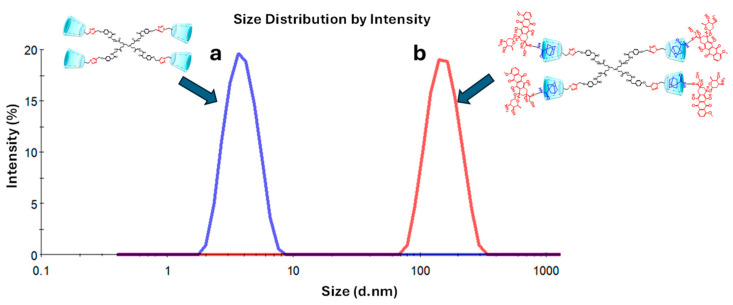
Size distribution of (**a**) PAMAM-βCD dendrimer and (**b**) IC PAMAM-βCD/Ad-h-Dox.

In previous research, it was described that one of the unique properties that characterizes dendrimers is their nanometric size, a similarity that is shared with proteins [37,38]. Specifically, the PAMAM dendrimer family presents diverse sizes that depend on their generation [39]. PAMAM dendrimers of large generations (G4–10) possess sizes between 4 and 14 nm, while small generations of dendrimers (G0–3) have a size smaller than 4 nm; for this reason, the latest dendrimers are not of interest to be studied as drug carriers [40,41,42]. Additionally, it was reported that the external size of βCD is ~0.78 nm [43,44]. The particle size distribution for the PAMAM–βCD dendrimer and the IC PAMAM-βCD/Ad-h-Dox is presented in Figure 4.

The results show that for the PAMAM–βCD dendrimer, an average particle size of 3.6 ± 1.1 nm is obtained (Figure 4a) with a polydispersity index (PDI) of 0.07, which is close in size to the PAMAM G3 but with the advantage that this new platform does have the capacity to encapsulate drugs even if it is a low-generation dendrimer. Additionally, the amine groups were removed from the periphery of the dendrimer because of their cellular toxicity [45]. Then, when the IC PAMAM-βCD/Ad-h-Dox was evaluated, an average particle size of 173.5 ± 44.0 nm (Figure 4b) and PDI of 0.325 were obtained. These results stand out from those obtained for the uncharged dendrimer, since this supramolecular assembly presents a larger particle size due to the presence of four prodrug molecules included in the βCD cavities. So, this difference in the particle size can be explained in function of the orientation in which the prodrug is assembled on the surface of the PAMAM-βCD dendrimer since the Ad ring completely enters the βCD cavity, causing residual Dox molecules to orient themselves towards the outside, producing a significant increase in the hydrodynamic diameter of the IC PAMAM-βCD/Ad-h-Dox [29]. Furthermore, the increase in the PDI value for the IC PAMAM-βCD/Ad-h-Dox could be related to the conformation adopted for the anthracycline rings of the Dox structure, which promote π-π stacking interactions between them, favoring the formation of aggregates that result in a significative increase in particle size for this supramolecular assembly [29].

#### 3.1.4. DSC and TGA

**Figure 5:**
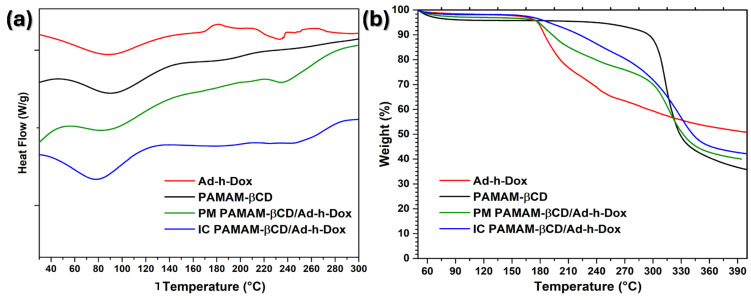
(**a**) DSC curves and (**b**) TGA thermograms of Ad-h-Dox, PAMAM-βCD dendrimer, PM PAMAM-βCD/Ad-h-Dox and IC PAMAM-βCD/Ad-h-Dox.

As a guest molecule is embedded into βCD cavities, a change is observed in the melting, dehydration and degradation temperatures, or they simply disappear [46]. Therefore, in the DSC curves (Figure 5a), an endothermic peak centered at 90 °C is observed for the three species, the Ad-h-Dox prodrug, PAMAM-βCD and PM PAMAM-βCD/Ad-h-Dox, which corresponds to moisture loss. Meanwhile, for the IC PAMAM-βCD/Ad-h-Dox, an endothermic peak centered at 80 °C for moisture loss is observed, which can be related to the loss of water molecules that surround the IC PAMAM-βCD/Ad-h-Dox which are not located in the βCD cavity. Regarding the Ad-h-Dox prodrug, an exothermic peak centered at 180 °C is observed, as a result of its crystallization. Moreover, an endothermic peak is observed at 230 °C that belongs to the melting point; the same transition temperatures are conserved for the PM PAMAM-βCD/Ad-h-Dox. For the PAMAM-βCD platform, another thermal transition is not observed at the maximum evaluation temperature (300 °C). Finally, in the IC PAMAM-βCD/Ad-h-Dox, an endothermic peak appears at 260 °C, corresponding to thermal degradation, which does not coincide with the degradation temperatures of the individual entities or their physical mixture.

In the TGA curves (Figure 5b), a 6% weight loss is observed for the Ad-h-Dox prodrug between 50 and 175 °C which corresponds to the loss of water. Then, the second stage goes from 175 to 320 °C, and a major weight loss can be observed which corresponds to the decomposition of this prodrug. For the PAMAM-βCD platform, a 10% weight loss is perceived between 50 and 295 °C, and a significant weight loss of 47% is observed between 295 and 340 °C which corresponds to the pyrolysis process of this platform. For the PM PAMAM-βCD/Ad-h-Dox, the same weight losses are maintained as those in the individual entities. Finally, for the IC PAMAM-βCD/Ad-h-Dox, a weight loss of 4% is observed between 50 and 160 °C which could be due to the loss of water and light volatile compounds. Subsequently, the IC PAMAM-βCD/Ad-h-Dox exhibits a 58% weight loss between 160 and 350 °C due to the decomposition and finally shows a continuous decomposition at a very slow rate after 350 °C. These results reinforce that the IC PAMAM/Ad-h-Dox was adequately prepared.

#### 3.1.5. SEM Analysis

**Figure 6:**
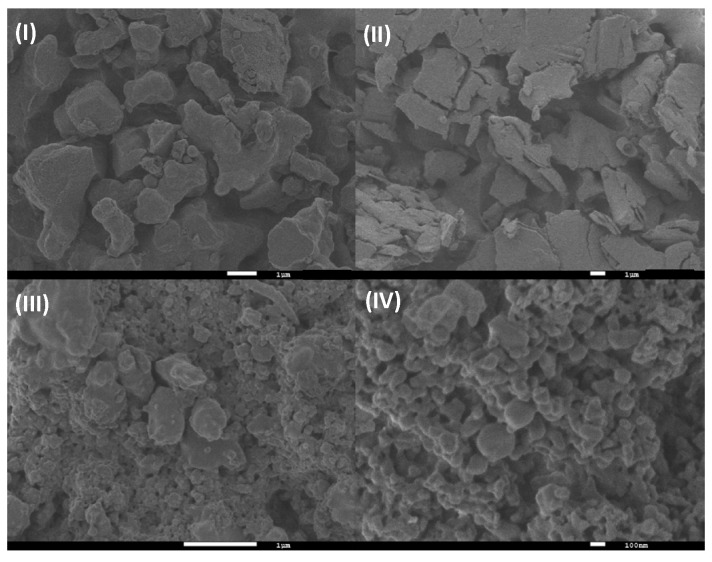
SEM photographs of (**I**) Ad-h-Dox, (**II**) PAMAM-βCD dendrimer, (**III**) PM of Ad-h-Dox and PAMAM-βCD dendrimer (1:1 molar ratio) and (**IV**) Ad-h-Dox/PAMAM-βCD IC.

For the SEM characterization, we started with the morphological analysis for the βCD/Ad-h-Dox assembly, and it was considered as a positive control to confirm the obtention of the PAMAM-βCD/Ad-h-Dox nanocarrier (see Figure S6 in SI). Then, in the same way, we analyzed the morphological changes presented by the solid form of the IC PAMAM-βCD/Ad-h-Dox and compared them with the characteristics of all the components used for the construction of this nanocarrier as follows: Ad-h-Dox (I), the PAMAM-βCD dendrimer (II), the PM (III) and the IC PAMAM-βCD/Ad-h-Dox (IV) (see Figure 6).

Firstly, the Ad-h-Dox prodrug (I) presents particles of rather irregular shape and size, which are typical of a non-crystalline solid, whereas the PAMAM-βCD dendrimer (II) is present in an amorphous state. The PAMAM-βCD/Ad-h-Dox (IV) assembly shows several globular morphologies that do not present their individual components, which corroborate the IC formation between the Ad-h-Dox prodrug with the internal βCD cavities [47]. Unlikely, the PM (III) presented Ad-h-Dox particles surrounding PAMAM-βCD dendrimer particles, which demonstrate the absence of any morphology related to IC formation. The different morphological constructions observed for the pure Ad-h-Dox prodrug, native PAMAM-βCD dendrimer and their IC confirmed that Ad-h-Dox is assembled into a βCD cavity. These observations confirmed the successful preparation of the PAMAM-βCD/Ad-h-Dox IC. Regarding the determination of particle size through SEM photographs, we found a distribution ≥ 200 nm. This increase in size is due to the big cluster aggregation between particles in the solid state, and this does not represent the real size of the individual PAMAM-βCD/Ad-h-Dox conjugates.

#### 3.1.6. In Vitro Release Study

**Figure 7:**
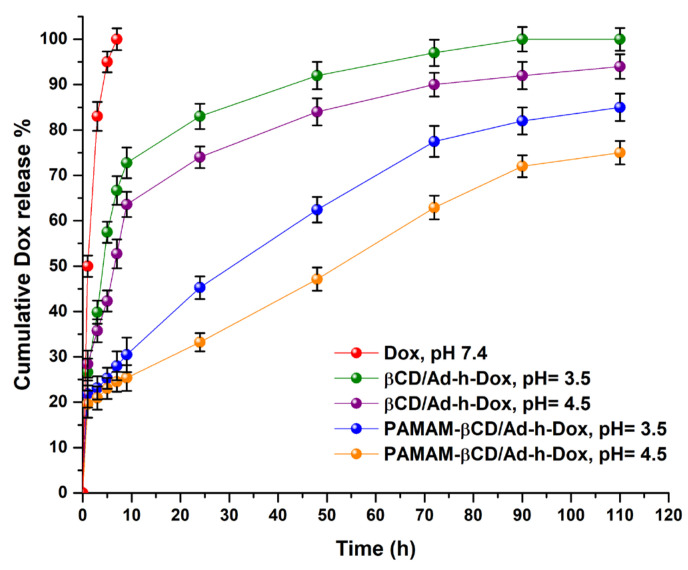
Cumulative drug release (%) of Dox from βCD/Ad-h-Dox IC and PAMAM-βCD/Ad-h-Dox nanocarrier in buffer solution (pH 3.5, 4.5 and 7.4) and pure Dox in pH = 7.4 at 37 °C during 110 h. (Mean ± SD; n = 3).

Release profiles for pure Dox (pH 7.4), Dox from βCD/Ad-h-Dox and the PAMAM-βCD/Ad-h-Dox nanocarrier at pH = 4.5 and 3.5 are shown in Figure 7.

The results show the observed behavior for each studied species by taking the free Dox as reference (graph in red), where it is noticeable that at pH = 7.4, the drug by itself diffuses rapidly without any type of control within 5 h. Then, in order to highlight the PAMAM-βCD/Ad-h-Dox nanocarrier design, the release profile for the native βCD/Ad-h-Dox assembly was analyzed, and in Figure 7, it is possible to distinguish that the release of Dox from this assembly is pH-dependent, and after a burst release during the first 10 h, the system reaches 80% at pH = 3.5 and 73% at pH = 4.5 of Dox release at 24 h (green and purple lines in the graph, respectively). After this time, a moderate release of Dox is observed, reaching 90% and 85% of drug release at 72 h at those same pH values. This behavior can be attributed to the fact that the hydrazone bond cleavable at acidic pH, when assembled in the internal cavity of βCD, is exposed to hydrolysis without any steric hindrance.

Additionally, the PAMAM-βCD/Ad-h-Dox nanocarrier maintains a pH-dependent release and shows a controlled and prolonged release of Dox. For this nanocarrier, an initial burst release is not observed, and up to 24 h, the system only releases 45% of drugs loaded at pH = 3.5 (graph blue) and 33% (graph orange) at pH = 4.5, in addition to 75% and 60% at 72 h at the aforementioned pH values. In this way, the PAMAM-βCD/Ad-h-Dox nanocarrier shows a controlled and prolonged Dox release, which indicates a great advance, since the Dox concentrations remain in an adequate range for more than 72 h. For this reason, we decided to explore the Dox release profile up to 110 h to verify that this trend observed in the initial times is preserved. This release profile behavior for the PAMAM-βCD/Ad-h-Dox nanocarrier can be attributed to the fact that in this design, the assembly is formed between the adamantane ring of the Ad-h-Dox prodrug and the βCD inner cavity, while outside the latter macrocycle and on the surface of the PAMAM-βCD dendrimer, the four anthracycline rings of the Dox structure are located which are sterically hindered, which would explain the absence of burst release. In addition, one of the hydrazone bonds cleavable at acidic pH probably becomes conformationally available for hydrolysis; thus, once a prodrug entity is cleaved, the other three become spatially more available for hydrolysis, and this adopted spatial distribution can explain the control in the release of Dox from the PAMAM-βCD/Ad-h-Dox nanocarrier. These results demonstrate that the PAMAM-βCD/Ad-h-Dox nanocarrier is a promising candidate as a controlled release system of Dox for cancer treatment.

#### 3.1.7. Cytotoxic Evaluation

As part of a systematic evaluation of the PAMAM-βCD/Ad-h-Dox nanocarrier, the in vitro cytotoxic activity was determined in a representative panel of cancer cell lines that are treated with Dox in the clinical field; the results are shown in Table 2. The obtained data are transcendental since they confirm the correct design of this novel Dox delivery system. Firstly, the nanocarrier PAMAM-βCD alone was evaluated, and it was found that it does not present cytotoxicity in any of the tested cell lines. In addition, the IC PAMAM-βCD/Ad-COOH was evaluated as a hydrolysis product that is formed after the delivery of Dox, with the aim of demonstrating that it is only when Dox is loaded in the nanosystem that it produces the expected biological effect, and neither the carrier nor its hydrolysis product contributes to cytotoxicity.

Consequently, as shown in Table 2, the PAMAM-βCD/AdCOOH assembly also does not present any cytotoxicity. Thus, when the Ad-h-Dox prodrug was tested, it showed moderate biological activity in all the analyzed cancer cell lines (see Table 2, entry 3). So, we decided to determine the cytotoxicity for the βCD/Ad-h-Dox supramolecular assembly (see entry 4 in Table 2), and it was found that the level of cytotoxic activity doubled (represented as a lower IC_50_ value), and even in the colorectal cancer cell line (SW-620), the cytotoxic activity level was four times higher for the βCD/Ad-h-Dox assembly. These first results demonstrate the importance of the Ad-h-Dox prodrug being loaded onto an appropriate carrier to show an improvement in cytotoxic activity. Finally, the IC_50_ values presented by the PAMAM-βCD/Ad-h-Dox nanocarrier are comparable to those presented by free Dox in practically all cell lines tested and particularly in breast cancer cell lines. These results are encouraging for the potential application of the PAMAM-βCD/Ad-h-Dox nanocarrier, since we can be assured with the certainty of quantitative evidence that this nanocarrier will present cytotoxic effects only when it has a loaded therapeutic agent. In addition, this design not only preserves the cytotoxic activity of Dox but also improves control in the load and delivery of the drug.

## 4. Conclusions

The structure of a novel PAMAM-βCD/Ad-h-Dox nanocarrier was characterized and confirmed by the ^1^H-NMR, 2D NMR NOESY, DLS, DSC, TGA and SEM techniques. It was demonstrated by ^1^H-NMR titration studies that the PAMAM-βCD nanocarrier can transport up to four units of Ad-h-Dox prodrugs. The cytotoxic activity results demonstrate that the nanocarrier alone and its hydrolysis product are innocuous compared to the PAMAM-βCD/Ad-h-Dox nanocarrier that showed cytotoxicity equivalent to that of free Dox in the tested cancer cell lines. The in vitro drug release assays for the PAMAM-βCD/Ad-h-Dox system showed an acidic pH-dependent behavior and a prolonged profile of up to more than 72 h, making this design a controlled and prolonged Dox release system for potential use in cancer treatment.

## Figures and Tables

**Table 1:** Diffusion coefficients in D_2_O for PAMAM-βCD nanocarrier, PAMAM-βCD/AdCOOH and PAMAM-βCD/Ad-h-Dox supramolecular assemblies measured by ^1^H DOSY NMR technique.

	Compounds		
	PAMAM-βCD	PAMAM-βCD/Ad-COOH ^a^	PAMAM-βCD/Ad-h-Dox
*D (10^−10^ m^2^/s)*	1.3	1.8	2.8

^a^ Data taken from Ref [26].

**Table 2:** Values of IC_50_ (μM) of molecules and their supramolecular assemblies evaluated ^a^.

	Cell line					
Molecule	HeLa	K-562	SK-LU-1	SW-620	MCF-7	MDA-MB-231
PAMAM-βCD	ND	ND	ND	ND	ND	ND
PAMAM-βCD/Ad-COOH	ND	ND	ND	ND	ND	ND
Ad-h-Dox	3.7 ± 0.5	17.3 ± 0.8	4.1 ± 0.5	10.3 ± 0.2	4.2 ± 0.7	6.7 ± 1.4
βCD/Ad-h-Dox	1.5 ± 0.6	3.8 ± 1.5	2.6 ± 1.5	2.6 ± 1.0	1.9 ± 0.4	4.2 ± 0.9
PAMAM-βCD/Ad-h-Dox	0.33 ± 0.04	1.0 ± 0.1	0.6 ± 0.1	1.0 ± 0.2	0.31 ± 0.02	1.3 ± 0.4
**Dox**	**0.19 ± 0.02**	**2.4 ± 0.4**	**0.24 ± 0.04**	**0.44 ± 0.04**	**0.20 ± 0.03**	**0.5 ± 0.2**

^a^ Data represent mean ± SD (n = 3). ND: not determined for concentrations used.

## Data Availability

The original contributions presented in the study are included in the article/supplementary material, further inquiries can be directed to the corresponding authors.

## Supplementary Materials

The following supporting information can be downloaded at https://www.mdpi.com/article/10.3390/pharmaceutics16121509/s1, Figure S1. Job plot for inclusion complex of Ad-h-Dox with PAMAM-βCD dendrimer; Figure S2. ^1^H-NMR spectra of βCD/Ad-h-Dox in D_2_O; Figure S3. Two-dimensional NMR NOESY spectrum of IC-βCD/Ad-h-Dox in D_2_O; Figure S4. ^1^H DOSY NMR in D_2_O (500 MHz) spectrum of PAMAM-βCD; Figure S5. ^1^H DOSY NMR in D_2_O (500 MHz) spectrum of PAMAM-βCD/Ad-h-Dox; Figure S6. SEM photographs of (I) Ad-h-Dox, (II) native βCD, (III) Ad-h-Dox and βCD physical mixture (1:1 molar ratio) and (IV) Ad-h-Dox/βCD inclusion complex

## References

[1] Chari R.V.J. (1998). Targeted delivery of chemotherapeutics: Tumor-activated prodrug therapy. Adv. Drug Deliv. Rev..

[2] Denny W.A. (2001). Prodrug strategies in cancer therapy. Eur. J. Med. Chem..

[3] Bisht S., Maitra A. (2009). Dextran–doxorubicin/chitosan nanoparticles for solid tumor therapy. WIREs Nanomed. Nanobiotechnology.

[4] Claessensa A.K.M., Ibragimovaa K.I.E., Geurtsa S.M.E., Bos M.E.M.M., Erdkamp F.L.G., Tjan-Heijnen V.C.G. (2020). The role of chemotherapy in treatment of advanced breast cancer: An overview for clinical practice. Crit. Rev. Oncol. Hematol..

[5] Nielsen D., Maare C., Skovsgaard T. (1996). Cellular resistance to anthracyclines. Gen. Pharmacol. Vasc. Syst..

[6] De Beer E.L., Bottone A.E., Voest E.E. (2001). Doxorubicin and mechanical performance of cardiac trabeculae after acute and chronic treatment: A review. Eur. J. Pharmacol..

[7] Aroui S., Brahim S., De Waard M., Kenani A. (2010). Cytotoxicity, intracellular distribution and uptake of doxorubicin and doxorubicin coupled to cell-penetrating peptides in different cell lines: A comparative study. Biochem. Biophys. Res. Commun..

[8] Šimůnek T., Štěrba M., Popelová O., Adamcová M., Hrdina R., Geršl V. (2010). Anthracycline-induced cardiotoxicity: Overview of studies examining the roles of oxidative stress and free cellular iron. Pharmacol. Rep..

[9] Octavia Y., Tocchetti C.G., Gabrielson K.L., Janssens S., Crijns H.J., Moens A.L. (2012). Doxorubicin-induced cardiomyopathy: From molecular mechanisms to therapeutic strategies. J. Mol. Cell Cardiol..

[10] Jiang W., Lionberger R., Yu L.X. (2011). In Vitro and In Vivo Characterizations of PEGylated Liposomal Doxorubicin. Bioanalysis.

[11] Shabbits J.A., Chiu G.N.C., Mayer L.D. (2002). Development of an in vitro Drug Release Assay That Accurately Predicts In Vivo Drug Retention for Liposome-Based Delivery Systems. J. Control Release.

[12] Szebeni J., Moghimi S.M. (2009). Liposome Triggering of Innate Immune Responses: A Perspective on Benefits and Adverse Reaction. J. Liposome Res..

[13] Kaminskas L.M., McLeod V.M., Porter C.J.H., Boyd B.J. (2012). Association of Chemotherapeutic Drugs with Dendrimer Nanocarriers: An Assessment of the Merits of Covalent Conjugation Compared to Noncovalent Encapsulation. Mol. Pharm..

[14] Maeda H. (2001). The enhanced permeability and retention (EPR) effect in tumor vasculature: The key role of tumor-selective macromolecular drug targeting. Adv. Enzym. Regul..

[15] Fang J., Nakamura H., Maeda H. (2011). The EPR effect: Unique features of tumor blood vessels for drug delivery, factors involved, and limitations and augmentation of the effect. Adv. Drug Deliv. Rev..

[16] Tolland O., Turrin C.O., Caminade A.-M., Majoral J.-P. (2009). Dendrimers and nanomedicine: Multivalency in action. New J. Chem..

[17] Wang H., Huang Q., Chang H., Xiao J., Cheng Y. (2016). Stimuli-responsive dendrimers in drug delivery. Biomater. Sci..

[18] Labieniec-Watala M., Watala C. (2015). PAMAM Dendrimers: Destined for Success or Doomed to Fail? Plain and Modified PAMAM Dendrimers in the Context of Biomedical Applications. J. Pharm. Sci..

[19] Jain K., Kesharwani P., Gupta U., Jain N.K. (2010). Dendrimer toxicity: Let’s meet the challenge. Int. J. Pharm..

[20] Luong D., Kesharwani P., Deshmukh R., Amin M.C.I.M., Gupta U., Greish K., Iyer A.K. (2016). PEGylated PAMAM dendrimers: Enhancing efficacy and mitigating toxicity for effective anticancer drug and gene delivery. Acta Biomater..

[21] Malik N., Wiwattanapatapee R., Klopsch R., Lorenz K., Frey H., Weener J.W., Meijer E.W., Paulus W., Duncan R. (2000). Dendrimers: Relationship between structure and biocompatibility in vitro, and preliminary studies on the biodistribution of 125 I-labelled polyamidoamine dendrimers in vivo. J. Control Release.

[22] Agrawal P., Gupta U., Jain N.K. (2007). Glycoconjugated peptide dendrimers-based nanoparticulate system for the delivery of chloroquine phosphate. Biomaterials.

[23] Namazi H., Adeli M. (2005). Dendrimers of citric acid and poly(ethylene glycol) as the new drug-delivery agents. Biomaterials.

[24] Hu J., Cheng Y., Ma Y., Wu Q., Xu T. (2009). Host-guest chemistry and physicochemical properties of the dendrimer-mycophenolic acid complex. J. Phys. Chem. B.

[25] Cheng Y., Xu T. (2008). The effect of dendrimers on the pharmacodynamic and pharmacokinetic behaviors of non-covalently or covalently attached drugs. Eur. J. Med. Chem..

[26] González-Méndez I., Hameau A., Laurent R., Bijnani C., Bourdon V., Caminade A.-M., Rivera E., Ching K.I.M.C. (2020). β-Cyclodextrin PAMAM Dendrimer: How to Overcome the Tumbling Process for Getting Fully Available Host Cavities. Eur. J. Org. Chem..

[27] Luo G.F., Xu X.D., Zhang J., Yang J., Gong Y.-H., Lei Q., Jia H.-Z., Li C., Zhuo R.-X., Zhang X.-Z. (2012). Encapsulation of an Adamantane-Doxorubicin Prodrug in pH-Responsive Polysaccharide Capsules for Controlled Release. ACS Appl. Mater. Interfaces.

[28] González-Méndez I., Dolores-Solano J., Porcu P., Ruiu A., Rojas-Aguirre Y., Rivera E. (2019). Optimized synthesis, characterization and in vitro systematic evaluation of adamantane-doxorubicin prodrugs sensitive to pH in breast cancer cells. J. Mol. Struct..

[29] González-Méndez I., Aguayo Ortíz R., Sorroza-Martínez K., Dolores-Solano J., Porcu P., Rivera E., Domínguez L. (2020). Conformational analysis by NMR and molecular dynamics of adamantane doxorubicin prodrugs and their assemblies with β-cyclodextrin: A focus on the design of platforms for controlled drug delivery. Bioorganic Med. Chem..

[30] Granadero D., Bordello J., Pérez-Alvite M.J., Novo M., Al-Soufi W. (2010). Host-guest complexation studied by fluorescence correlations pectroscopy: Adamantane-cyclodextrin inclusion. Int. J. Mol. Sci..

[31] Harries D., Rau D.C., Parsegian V.A. (2005). Solutes probe hydration in specific association of cyclodextrin and adamantane solutes. J. Am. Chem. Soc..

[32] Sheng J., Wang Y., Xiong L., Luo Q., Li X., Shen Z., Zhu W. (2017). Injectable doxorubicin-loaded hydrogels based on dendron-like β-cyclodextrin–poly(ethylene glycol) conjugates. Polym. Chem..

[33] Kasprzak A., Koszytkowska-Stawińska M., Nowicka A.M., Buchowicz W., Poplawska M. (2019). Supramolecular Interactions between β-Cyclodextrin and the Nucleobase Derivatives of Ferrocene. J. Org. Chem..

[34] Ferrazza R., Rossi B., Guella G. (2014). DOSY-NMR and Raman Investigations on the Self-Aggregation and Cyclodextrin Complexation of Vanillin. J. Phys. Chem. B.

[35] Schneider H.-J., Hacket F., Rüdiger V., Ikeda H. (1998). NMR Studies of Cyclodextrins and Cyclodextrin Complexes. Chem. Rev..

[36] Kasprzak A., Dabrowski B., Zuchowska A. (2020). A biocompatible poly(amidoamine) (PAMAM) dendrimer octa-substituted with α-cyclodextrin towards the controlled release of doxorubicin hydrochloride from its ferrocenyl prodrug. RSC Adv..

[37] Tomalia D.A. (2005). Birth of a New Macromolecular Architecture: Dendrimers as Quantized Building Blocks for Nanoscale Synthetic Polymer Chemistry. Prog. Polym. Sci..

[38] Esfand R., Tomalia D.A. (2001). Poly(Amidoamine) (PAMAM) Dendrimers: From Biomimicry to Drug Delivery and Biomedical Applications. Drug Discov. Today.

[39] Svenson S., Tomalia D.A. (2005). Dendrimers in Biomedical Applications—Reflections on the Field. Adv. Drug Deliv. Rev..

[40] Patel M., De Paoli S.H., Elhelu O.K., Farooq S., Simak J. (2019). Cell Membrane Disintegration and Extracellular Vesicle Release in a Model of Different Size and Charge PAMAM Dendrimers Effects on Cultured Endothelial Cells. Nanotoxicology.

[41] Aftab S., Shah A., Nadhman A., Kurbanoglu S., Aysıl Ozkan S., Dionysiou D.D., Shukla S.S., Aminabhavi T.M. (2018). Nanomedicine: An Effective Tool in Cancer Therapy. Int. J. Pharm..

[42] Berényi S., Mihály J., Wacha A., Toke O., Bóta A. (2014). A Mechanistic View of Lipid Membrane Disrupting Effect of PAMAM Dendrimers. Colloids Surf. B Biointerfaces.

[43] Crini G. (2014). Review: A History of Cyclodextrins. Chem. Rev..

[44] Crini G., Fourmentin S., Fenyvesi É., Torri G., Fourmentin M., Morin-Crini N. (2018). Cyclodextrins, from Molecules to Applications. Environ. Chem. Lett..

[45] Fox L.J., Richardson R.M., Briscoe W.H. (2018). PAMAM Dendrimer—Cell Membrane Interactions. Adv. Colloid Interface Sci..

[46] Abarca R.L., Rodríguez F.J., Guarda A., Galotto M.J., Bruna J.E. (2016). Characterization of Beta-Cyclodextrin Inclusion Complexes Containing an Essential Oil Component. Food Chem..

[47] Yousef T., Hassan N., Akbar E.A. (2015). Synthesis of the Dendritic Type beta-Cyclodextrin on Primary Face via Click Reaction Applicable as Drug Nanocarrier. Carbohydr. Polym..

