# Supplementary material for: Using Poly(amidoamine) PAMAM-βCD Dendrimer for Controlled and Prolonged Delivery of Doxorubicin as Alternative System for Cancer Treatment
Source: Pharmaceutics. 2024 Nov 23;16(12):1509. doi: 10.3390/pharmaceutics16121509 (PMC11728618; doi:10.3390/pharmaceutics16121509)
Supplement: Supplementary file 1 [file pharmaceutics-16-01509-s001.zip › pharmaceutics-3273330-supplementary.pdf]

Using a poly(amidoamine) PAMAM-βCD dendrimer for controlled and prolonged delivery of doxorubicin as alternative system for cancer treatment

Kendra Sorroza-Martínez [1], Ignacio González-Sánchez [2], Raúl Villamil-Ramos [3], Marco Cerbón [2], Jorge Antonio Guerrero-Álvarez [3], Cristina Coronel-Cruz [4], Ernesto Rivera [5,*] and Israel González-Méndez [3,].**

Supplementary Material

Figure S1. Job plot for the inclusion complex of Ad-h-Dox with PAMAM-βCD dendrimer p. 2

Figure S2. ^{1}H-NMR spectra of βCD/Ad-h-Dox in D_2O p. 2

Figure S3. 2D NMR NOESY spectrum of IC-βCD/Ad-h-Dox in D_2O p. 3

Figure S4. ^{1}H DOSY NMR in D_2O (500 MHz) spectrum of PAMAM-βCD p. 3

Figure S5. ^{1}H DOSY NMR in D_2O (500 MHz) spectrum of PAMAM-βCD/Ad-h-Dox p. 4

Figure S6. SEM photographs of (**I**) Ad-h-Dox, (**II**) native βCD, (**III**) Ad-h-Dox and βCD physical mixture (1:1 molar ratio) and (**IV**) Ad-h-Dox/βCD inclusion complex p. 4

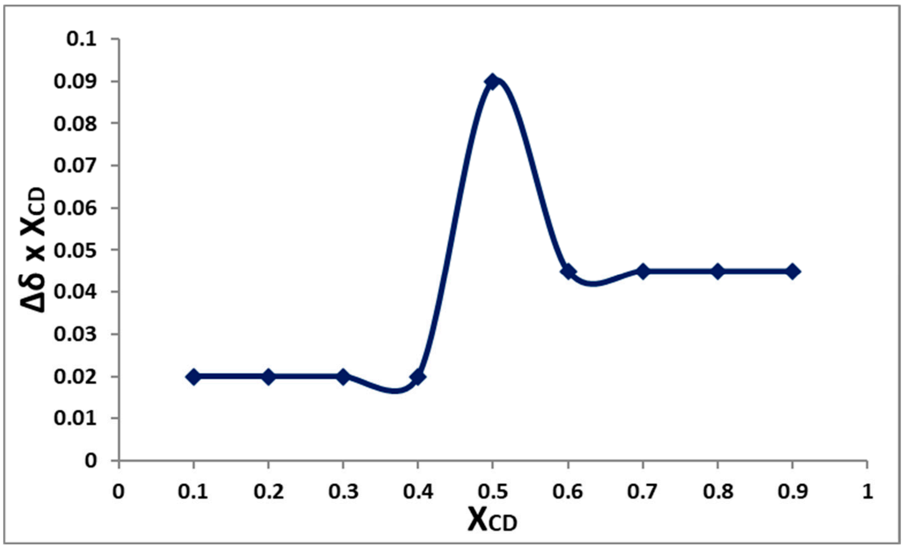

Figure S1. Job plot for the inclusion complex of Ad-h-Dox with PAMAM-βCD dendrimer, [βCD] + [Ad-h-Dox] = 2.5 mM at 298 K, in D_2O. (The inflection point was found to be at 0.5, which confirms the stoichiometry 1:1.)

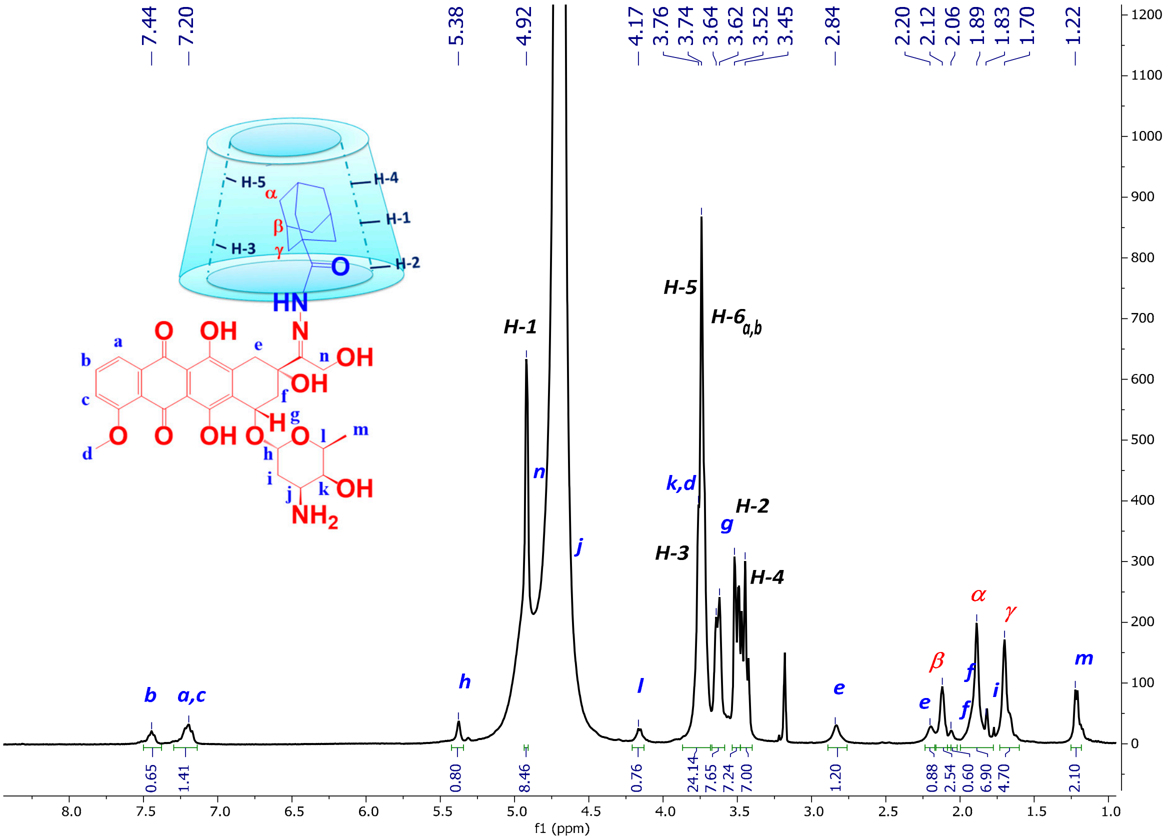

Figure S2. ^{1}H-NMR spectra of βCD/Ad-h-Dox in D_2O.

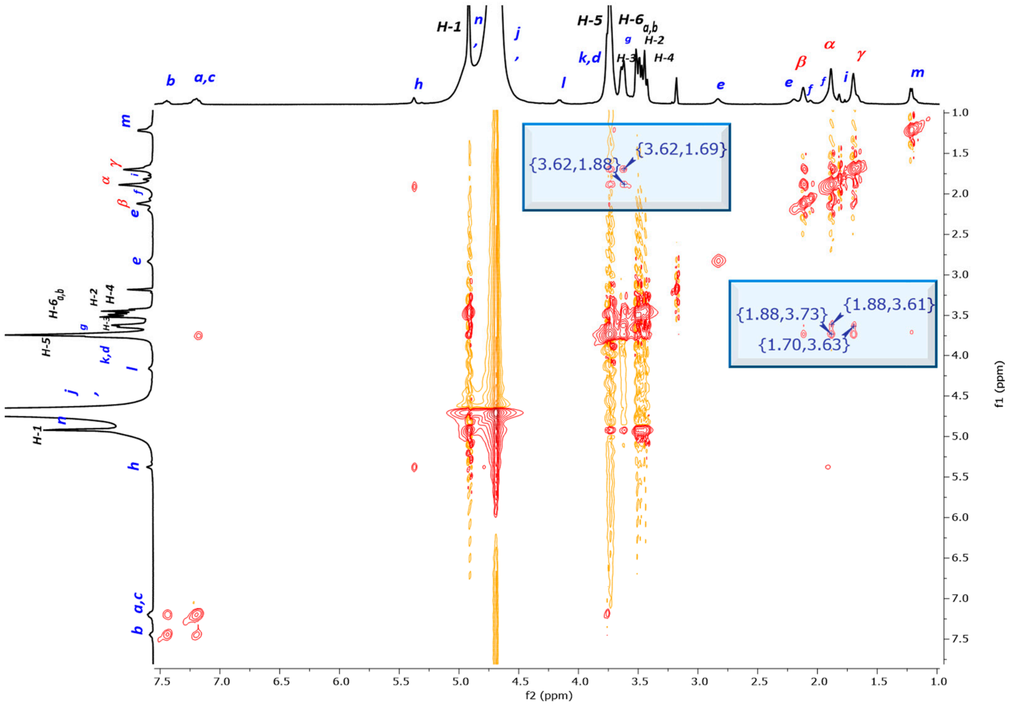

Figure S3. 2D NMR NOESY spectrum of IC-βCD/Ad-h-Dox in D_2O (highlighted in blue the interaction between Ad residues with inner cavity in βCD unit).

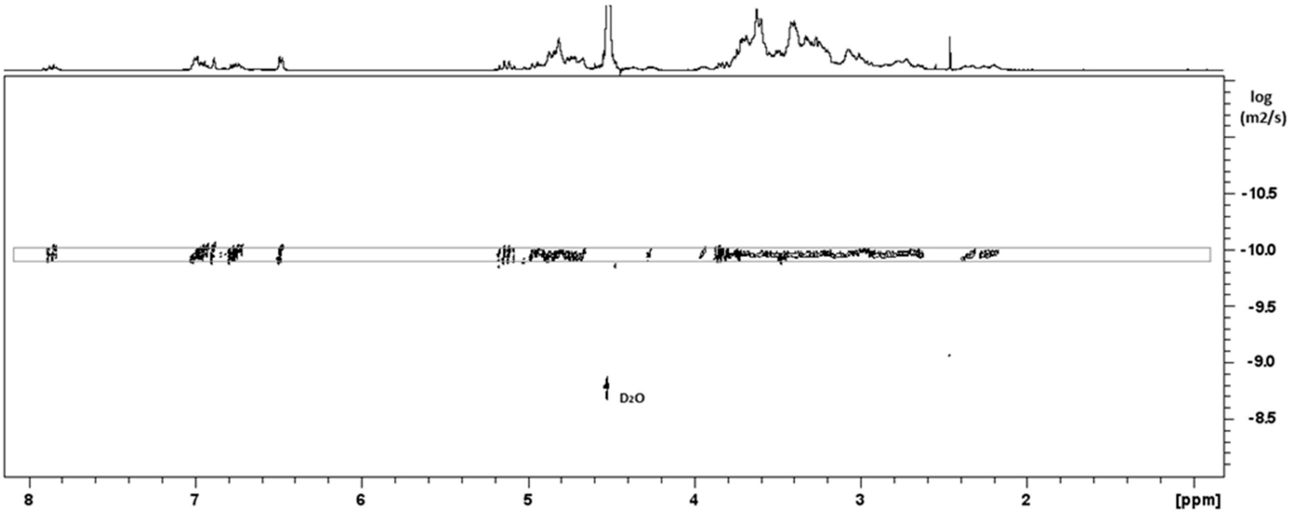

Figure S4. ^1H DOSY NMR in D_2O (500 MHz) spectrum of PAMAM-βCD.

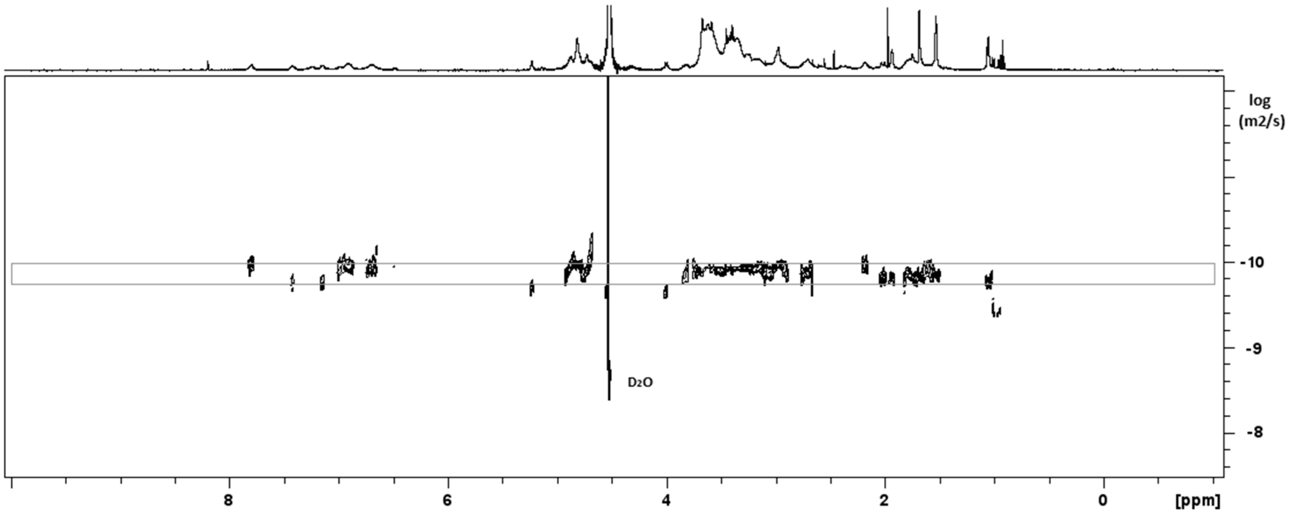

Figure S5. ^{1}H DOSY NMR in D_2O (500 MHz) spectrum of PAMAM-βCD/Ad-h-Dox.

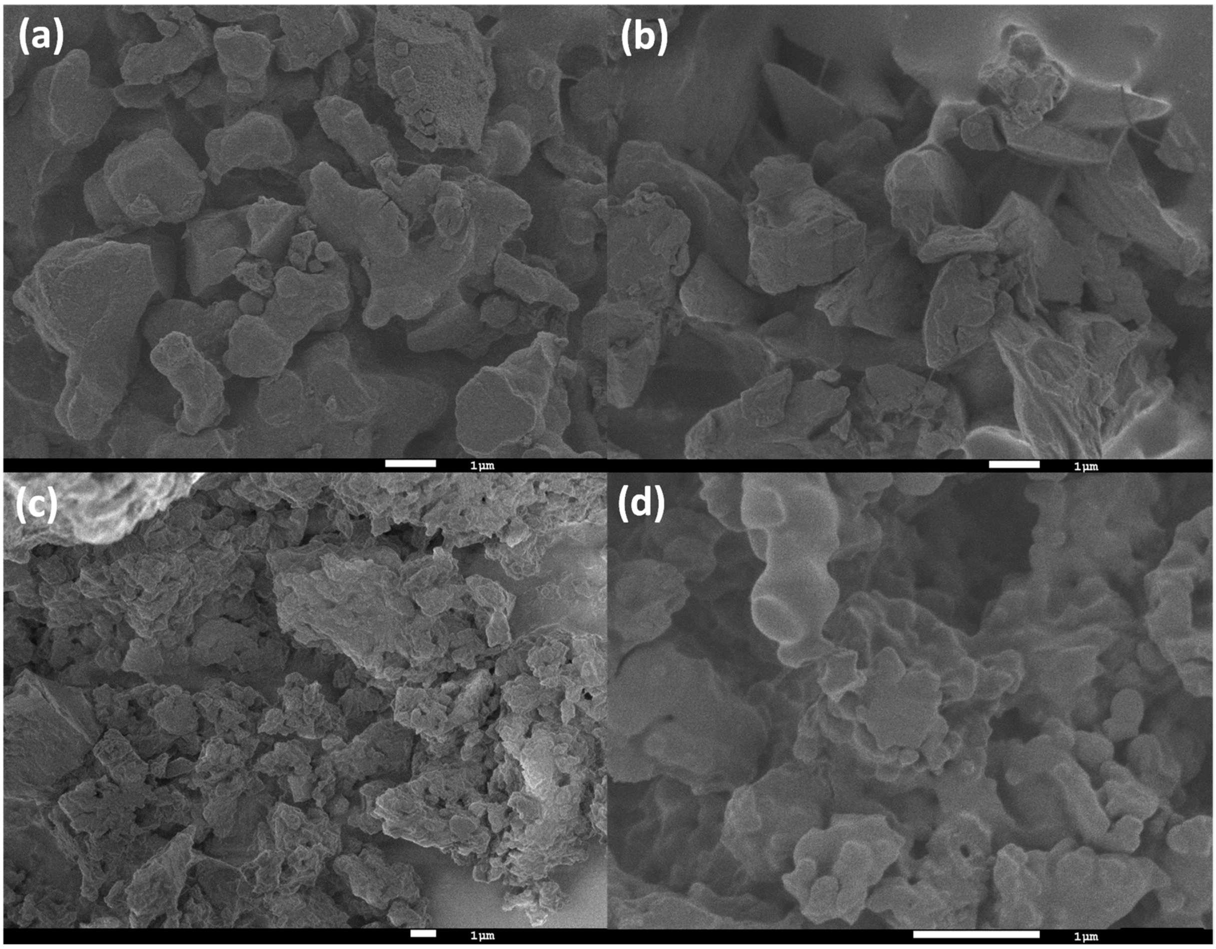

Figure S6. SEM photographs of (**a**) Ad-h-Dox, (**b**) native βCD, (**c**) Ad-h-Dox and βCD physical mixture (1:1 molar ratio) and (**d**) Ad-h-Dox/βCD inclusion complex.
